# AmiD Is a Novel Peptidoglycan Amidase in *Wolbachia* Endosymbionts of *Drosophila melanogaster*

**DOI:** 10.3389/fcimb.2017.00353

**Published:** 2017-08-04

**Authors:** Miriam Wilmes, Kirstin Meier, Andrea Schiefer, Michaele Josten, Christian F. Otten, Anna Klöckner, Beate Henrichfreise, Waldemar Vollmer, Achim Hoerauf, Kenneth Pfarr

**Affiliations:** ^1^Institute of Medical Microbiology, Immunology and Parasitology, University Hospital Bonn Bonn, Germany; ^2^Institute for Cell and Molecular Bioscience, Newcastle University Newcastle upon Tyne, United Kingdom; ^3^Institute for Pharmaceutical Microbiology, University of Bonn Bonn, Germany; ^4^German Center for Infection Research (DZIF), Partner Site Bonn-Cologne Bonn, Germany

**Keywords:** metalloenzyme, amidase, peptidoglycan, lipid II, *Wolbachia*, host recognition

## Abstract

*Wolbachia* endobacteria are obligate intracellular bacteria with a highly reduced genome infecting many arthropod and filarial species, in which they manipulate arthropod reproduction to increase their transmission and are essential for nematode development and survival. The *Wolbachia* genome encodes all enzymes required for the synthesis of the cell wall building block lipid II, although a peptidoglycan-like structure has not been detected. Despite the ability to synthesize lipid II, *Wolbachia* from arthropods and nematodes have only a subset of genes encoding enzymes involved in the periplasmic processing of lipid II and peptidoglycan recycling, with arthropods having two more than nematodes. We functionally analyzed the activity of the putative cell wall hydrolase AmiD from the *Wolbachia* endosymbiont of *Drosophila melanogaster*, an enzyme not encoded by the nematode endobacteria. *Wolbachia* AmiD has Zn^2+^-dependent amidase activity and cleaves intact peptidoglycan, monomeric lipid II and anhydromuropeptides, substrates that are generated during bacterial growth. AmiD may have been maintained in arthropod *Wolbachia* to avoid host immune recognition by degrading cell wall fragments in the periplasm. This is the first description of a wolbachial lipid II processing enzyme putatively expressed in the periplasm.

## Introduction

The genus *Wolbachia* represents a group of obligate intracellular Gram-negative bacteria that are widespread in arthropods and filarial nematodes. Some species of the latter infect humans and cause lymphatic filariasis (lymphedema; hydrocele) or onchocerciasis (river blindness) (Specht et al., 2013). *Wolbachia* endobacteria reside in host-derived vacuoles and are vertically transmitted from females to their offspring. The interaction of *Wolbachia* with their hosts ranges from mutualistic symbiosis to parasitism (Werren et al., 2008). In filaria, these endobacteria are required for worm development, fertility and survival, and thus filariasis can be effectively treated with antibiotics targeting *Wolbachia* (Taylor and Hoerauf, 2001; Taylor et al., 2010). *Wolbachia* in arthropods are largely parasitic and manipulate host reproduction by several mechanisms including feminization of genetic males or cytoplasmic incompatibility to increase the number of infected females in the population (Werren et al., 2008). However, there are exceptions and in some cases the arthropod host requires *Wolbachia* for oogenesis and positive benefits to fitness have also been demonstrated in terms of resistance to different pathogens and in nutrient provisioning (Zug and Hammerstein, 2015). For example, in the bedbug *Cimex lectularis, Wolbachia* were shown to be essential for growth and reproduction of the host by providing B vitamins, indicating a mutualistic relationship (Nikoh et al., 2014).

A characteristic feature of intracellular bacteria is a highly reduced genome due to adaption to the host (Stepkowski and Legocki, 2001). The *Wolbachia* genomes (ranging from 0.9 to 1.5 Mb) have lost many genes, particularly those involved in biosynthetic pathways (Wu et al., 2004; Foster et al., 2005; Lindsey et al., 2016). However, the genomes of these endobacteria encode all enzymes required for the synthesis of the cell wall precursor lipid II although a functional cell wall has not been detected. Intracellular bacteria do not need a cell wall for osmotic stabilization, but it has been shown that lipid II is crucial in *Wolbachia* for coordinated cell division (Vollmer et al., 2013).

The major component of bacterial cell walls is peptidoglycan (PG), a polymer consisting of glycan strands with alternating units of N-acetylglucosamine (Glc*N*Ac) and N-acetylmuramic acid (Mur*N*Ac) that are connected via peptides attached by an amide linkage to the lactyl group of Mur*N*Ac. The peptides of Gram-negative bacteria typically contain L-alanine, D-glutamate, meso-diaminopimelic acid (mDAP) and D-alanine (Typas et al., 2012).

PG biosynthesis is a multi-step process involving reactions in the cytoplasm and both sides of the cytoplasmic membrane. It starts in the cytoplasm with assembly of the soluble cell wall precursors UDP-Glc*N*Ac and UDP-Mur*N*Ac-pentapeptide. These precursors and the membrane carrier undecaprenyl phosphate are utilized to produce lipid II [undecaprenyl-pyrophosphoryl-Mur*N*Ac-(pentapeptide)-Glc*N*Ac] at the inner leaflet of the cytoplasmic membrane. Lipid II is then translocated across the membrane and incorporated into the growing peptidoglycan network by glycosyltransferase and transpeptidation reactions (Typas et al., 2012).

Cell wall hydrolases such as lytic transglycosylases, endopeptidases and amidases are capable of cleaving different bonds within the net-like peptidoglycan structure. They allow for the separation of daughter cells at the end of cell division and release turnover products during cell growth, which are subsequently translocated into the cytoplasm via permeases and recovered by several recycling enzymes (Vollmer et al., 2008; Johnson et al., 2013). Lytic transglycosylases cleave the glycosidic bond between Mur*N*Ac and Glc*N*Ac units, amidases and endopeptidases hydrolyze various amide bonds in the PG (Vollmer et al., 2008).

*Escherichia coli* produces five N-acetylmuramoyl-L-alanine amidases. The periplasmic amidases AmiA, AmiB and AmiC are involved in septum cleavage during cell division and can substitute for each other in function (Heidrich et al., 2001; Priyadarshini et al., 2007). The cytoplasmic AmpD participates in the recycling of PG fragments and uses only anhydromuropeptides as a substrate, which are characterized by a glycosidic bond between C1 and C6 of Mur*N*Ac as the result of glycan chain cleavage by lytic transglycosylases (Jacobs et al., 1995). The lipoprotein AmiD is anchored to the outer membrane and has a broad substrate spectrum, but its precise role is unknown (Uehara and Park, 2007). Other Gram-negative bacteria encode only a single periplasmic N-acetylmuramoyl-L-alanine amidase. In *Neisseria gonorrhoeae* AmiC functions in cell separation and PG fragment release (Lenz et al., 2016), while in *Vibrio cholerae* AmiB is crucial for cell division and growth (Möll et al., 2014). In intracellular pathogens of the genus *Chlamydia*, AmiA has, in contrast to free-living bacteria, a dual enzymatic activity acting as an N-acetylmuramoyl-L-alanine amidase and a DD-carboxypeptidase (Klöckner et al., 2014).

**Figure 1:**
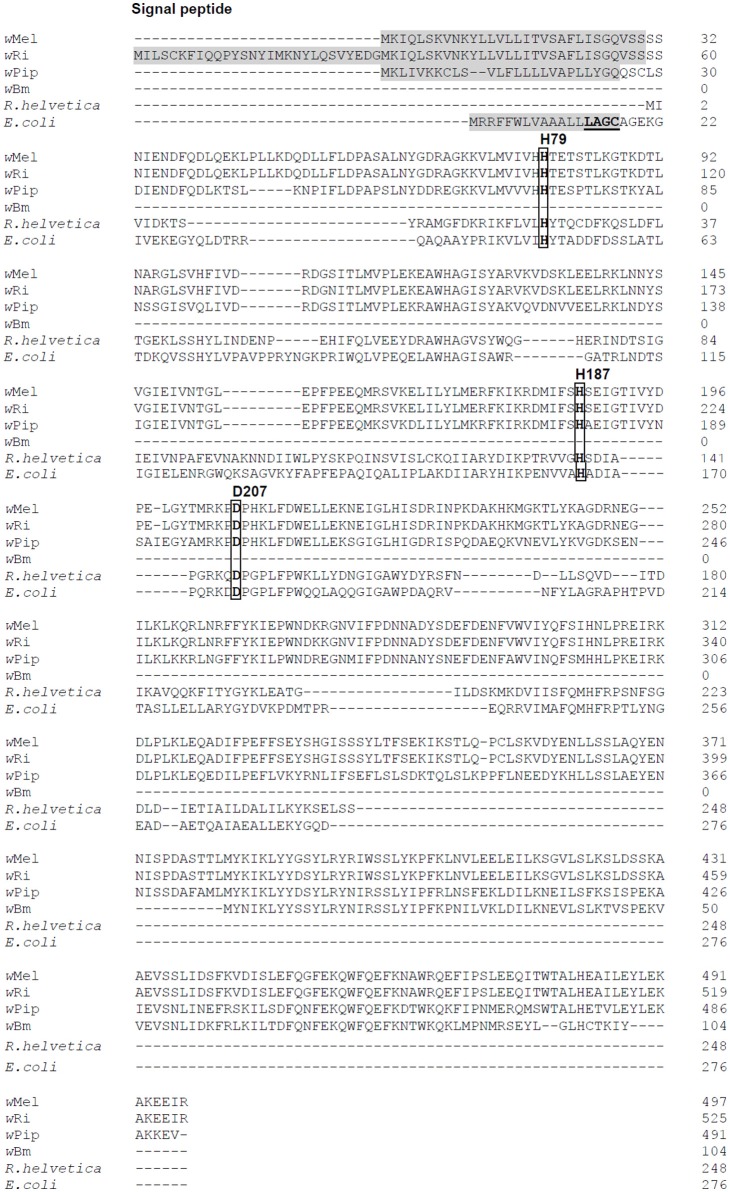
Amino acid sequence alignment of AmiD from *E. coli* and selected *Wolbachia* species. Four *Wolbachia* AmiD sequences (*w*Mel, *Wolbachia* from *Drosophila melanogaster*, WD1073; *w*Ri, *Wolbachia* from *Drosophila simulans*, EAL60110; *w*PiP, *Wolbachia* from *Culex quinquefasciatus* Pel, CAQ54941; *w*Bm, *Wolbachia* from *Brugia malayi*, AAW71270) and one Rickettsia species (*R. helvetica*, WP_010420485.1) were aligned with that of *E. coli* (NP_415388).The signal peptides of *Wolbachia* AmiD (shaded in gray) were predicted by SignalP (http://www.cbs.dtu.dk/services/SignalP/). The *E. coli* AmiD cysteine-containing lipobox motif at the C-terminal end of the signal sequence is underlined. The amidase active site of *E. coli* AmiD contains three zinc-coordinating residues (H50, H166, D176). These active site residues (boxed) are conserved among insect *Wolbachia* (in *w*Mel: H79, H187, D207).

The genome of *Wolbachia* from *Drosophila melanogaster* (*w*Mel) has retained only one predicted periplasmic cell wall hydrolase (WD1073). Sequence alignments with periplasmic *E. coli* AmiA, AmiB, AmiC and AmiD reveal the highest homology to *E. coli* AmiD (27% sequence identity). Sequenced genomes of filarial *Wolbachia* residing in *Brugia malayi* (*w*Bm) and *Onchocerca volvulus* (*w*Ov) show that these strains have lost the ability to synthesize any of these enzymes, e.g., *w*Bm0682 might encode an amidase in *w*Bm, but genome analysis has concluded that it is a pseudogene (Figure 1; Wu et al., 2004; Foster et al., 2005).

In this study, we analyzed the activity of AmiD from *w*Mel. Our results demonstrate that recombinantly expressed AmiD from *Wolbachia* (AmiD^wol^) cleaves the Mur*N*Ac-L-Ala bond in PG, monomeric lipid II and anhydromuropeptides. The amidase activity is Zn^2+^-dependent and inhibited in the presence of the metal chelators EDTA and 1,10-phenanthroline. The enzymatic activity of AmiD^wol^ may have a crucial role in cleavage of a PG-like structure and allow *Wolbachia* to avoid host organism immune responses by degrading cell wall fragments in the periplasm that could be recognized by innate immune receptors (Buchon et al., 2014).

## Materials and methods

### *In silico* analysis of AmiD^wol^ localization

We used BLAST analysis (http://blast.ncbi.nlm.nih.gov/Blast.cgi) to identify sequences similar to WD1073 and the Clustal Omega multiple sequence alignment tool (http://www.ebi.ac.uk/Tools/msa/clustalo/) to align AmiD sequences of different species. A signal peptide for periplasmic secretion and cleavage site was predicted by SignalP (http://www.cbs.dtu.dk/services/SignalP-3.0/).

### Cloning and expression of AmiD^wol^

The gene *amiD* from *w*Mel was amplified without its native signal sequence using the primers listed in Table 1. The PCR product was cloned into the expression vector pASK-IBA2C (IBA Lifesciences, Göttingen, Germany) using the *BsaI* restriction sites in the primers to generate a protein with an N-terminal OmpA leader peptide for periplasmic secretion and a C-terminal Strep-tag for purification. To test whether AmiD^wol^ is secreted into the periplasm, *amiD* from *w*Mel was cloned with its native signal sequence into the cytoplasmic expression vector pASK-IBA3plus (IBA Lifesciences) using the *BsaI* restriction sites.

**Table 1:** Primers used to clone *amiD* into expression vectors and for mutagenesis of active site residues.

**Primer name**	**Sequence (5′ → 3′)**
IBA2C_amiD-for_2	ATGGTA**GGTCTC**AGGCCTCAAGCAATATCGAGAATGATTTTCA
IBA2C_amiD-rev_2	ATGGTA**GGTCTC**AGCGCTACGAATTTCTTCCTTTGCTTTTTCTA
amiD_mut3-for (H79A)	GGTTATAGTTCACGCGACTGAAACATCAAC
amiD_mut3-rev (H79A)	GTTGATGTTTCAGTCGCGTGAACTATAACC
amiD_mut5-for (D207A)	GCTATACAATGCGTAAACCAGCGCCACACAAATTGTTTGATTG
amiD_mut5-rev (D207A)	CAATCAAACAATTTGTGTGGCGCTGGTTTACGCATTGTATAGC
IBA3_amiD-for	ATGGTA**GGTCT**CAAATGATGAAAATCCAACTATCTAAAGTCAAC
IBA3_amiD-rev	ATGGTA**GGTCTC**AGCGCTACGAATTTCTTCCTTTGCTTTTTCTA

*BsaI restriction sites are written in bold. Mutated positions are underlined*.

### Site-directed mutagenesis

H79 and D207 in AmiD^wol^ were changed to alanine using the QuikChange Lightning Site-Directed Mutagenesis Kit (Agilent Technologies, Waldbronn, Germany) according to the manufacturer's instructions. Primers are listed in Table 1. Correct base changes were confirmed by sequencing (SeqLab, Göttingen, Germany).

### Co-solvent assisted overproduction and purification of AmiD^wol^

*E. coli* JM83 (DSM3947) containing pASK-IBA2C_amiD or pASK-IBA2C (empty vector) was grown in 2 L salt-free LB medium containing 342 mM D-mannitol (equivalent to 342 osm/L/10 g/L sodium chloride) and 30 μg/mL chloramphenicol at 25°C (Otten et al., 2015). Expression of AmiD^wol^ was induced at OD_600_ 0.6 by addition of 200 ng/mL anhydrotetracycline (AHT; IBA Lifesciences, Göttingen, Germany) and cells were incubated at 25°C until they started lysing due to *in vivo* activity of AmiD^wol^.

Purification of Strep-tagged proteins was performed according to the manufacturer's instructions for periplasmic expression of metalloproteins (IBA Lifesciences). Cell pellets were suspended in 10 mL buffer P (100 mM Tris-HCl, pH 8; 500 mM sucrose; 2 mg/mL polymyxin B sulfate; 1 mg/mL lysozyme) and incubated for 30 min on ice. To reduce viscosity, benzonase (20 U/mL) was added and the suspension was incubated for 15 min on ice. Cleared lysate containing the Strep-tagged proteins was prepared by centrifugation (38,800 g, 15 min, 4°C).

Proteins were purified by gravity flow chromatography at 4°C. One mL Strep-Tactin® Sepharose (IBA Lifesciences) was applied to a 1 mL polypropylene column and equilibrated with 2 mL buffer W (100 mM Tris-HCl, pH 8; 150 mM NaCl). The cleared lysate was loaded onto the column and washed five times with 1 mL buffer W. Finally, AmiD^wol^ was eluted six times with 0.5 mL buffer E (100 mM Tris-HCl, pH 8; 150 mM NaCl, 2.5 mM desthiobiotin). AmiD^wol^ was stored in 50% glycerol at −20°C and was stable for at least 6 months.

### Growth kinetics

*E. coli* JM83 harboring periplasmic expression vectors was grown in LB medium supplemented with 30 μg/mL chloramphenicol at 25°C and the OD_600_ was measured every 30 min. AmiD^wol^ expression was induced with 200 ng/mL AHT at an OD_600_ of 0.6.

### Lipid II synthesis

Lipid II was synthesized *in vitro* as described previously (Schneider et al., 2004). Briefly, isolated membranes from *Micrococcus flavus* (Schneider et al., 2004) were incubated with UDP-Mur*N*Ac-pentapeptide from *Staphylococcus simulans* 22 or *Bacillus cereus* T (Schneider et al., 2004), C_55_-P (Larodan, Solna, Sweden) and UDP-Glc*N*Ac (Sigma-Aldrich, Taufkirchen, Germany). Optimal conditions had to be titrated in an analytical assay before preparing lipid II in a larger scale. Lipid II was obtained by mixing 20–30 μl isolated membranes, 7.5–15 μl UDP-Mur*N*Ac-pentapeptide, 5 nmol C_55_-P, 1 mM UDP-Glc*N*Ac, 5 mM MgCl_2_, 60 mM Tris (pH 7.5), 0.5% Triton X-100 (v/v) in a final volume of 75 μl. The mixture was incubated for 4 h at 30°C with shaking and afterwards extracted and visualized by thin layer chromatography (TLC) as described previously (Schneider et al., 2004; Klöckner et al., 2014). Lipid II synthesis in a preparative scale was achieved by using a 200-fold volume of the analytical scale.

Purification of lipid II was performed on a 5 mL HiTrap DEAE FF column (GE Healthcare, Freiburg, Germany) and eluted with a linear gradient of chloroform/methanol/water (2:3:1, v/v) to chloroform/methanol/300 mM ammonium bicarbonate (2:3:1, v/v). Lipid II was quantified by measuring the released phosphate upon total hydrolysis (Rouser et al., 1970).

### AmiD^wol^ activity against PG or lipid II

Preparation and staining of sacculi with Remazol Brilliant Blue (RBB) using *E. coli* W3110 (DSM3947) was performed as described previously (Uehara et al., 2009, 2010). For the dye-release assays, 20 μL of stained PG sacculi were incubated at 30°C overnight with 4 μM of purified AmiD^wol^ in a final volume of 200 μL containing 50 mM Tris (pH 7.5) and 5% dimethyl sulfoxide (DMSO). Samples were centrifuged [20,000 g, 20 min, room temperature (RT)] and absorbance of the supernatants was measured at 595 nm.

Using lipid II as a substrate for activity assays, 4 μg purified AmiD^wol^, 2 nmol lipid II, 50 mM Tris (pH 7.5) and 5% DMSO in a volume of 40 μL were incubated for 4 h at 30°C. Reaction products were extracted with 40 μL of n-butanol/pyridine acetate (2:1 v/v, pH 4.2), centrifuged (21,000 g, 5 min, RT) and analyzed by TLC and mass spectrometry (MS) as described previously (Klöckner et al., 2014).

To inhibit amidase activity of AmiD^wol^, 1 mM EDTA was added to buffer P, buffer W and buffer E during lysis and purification of the protein according to the manufacturer's instructions (IBA Lifesciences). 5 mM 1,10-phenanthroline (Sigma-Aldrich) was added to the reaction mixtures of the activity tests described above.

### Cleavage of anhydromuropeptides

PG sacculi from *E. coli* were prepared as described (Glauner, 1988). The PG (750 μg) was digested with the lytic transglycosylase Slt (1 μM) in a final volume of 210 μL containing 10 mM HEPES (pH 7.5) and 150 mM NaCl for 18 h at 37°C. The reaction mixture was heated for 10 min at 100°C and centrifuged at 17,000 g for 20 min. The supernatant containing the 1,6-anhydro-muropeptides was collected and stored at 2–8°C.

1,6-Anhydro-muropeptides (15 μL) were incubated with AmiD^wol^ (2 μM) in a final volume of 50 μL containing 50 mM Tris (pH 7.5) and 5% DMSO for 4 h at 30°C. Samples were boiled for 10 min, centrifuged for 20 min and the supernatant recovered. The pH of the supernatant was adjusted to pH 4 with 20% phosphoric acid. HPLC analysis was carried out as described (Glauner, 1988), and selected peaks were collected and analyzed by mass spectrometry (Bui et al., 2009).

### Mass spectrometry

For detection of reaction products from AmiD^wol^ activity assays using lipid II, 1 μL of the sample was placed onto a ground steel matrix assisted laser desorption ionization time-of-flight target plate (Bruker Daltonik, Bremen, Germany) and allowed to dry at RT. Each sample was then overlaid with 1 μL of matrix (saturated solution of 6-Aza-2-thiothymine in 50% ethanol/20 mM diammonium citrate or alpha-cyano-cinnamic acid in 33% acetonitrile/0.1% trifluoroacetic acid) and air dried at RT. Spectra were recorded in the reflector negative mode on a Biflex III mass spectrometer (Bruker Daltonik).

### Statistical analysis

For statistical analysis, GraphPad Prism 5 software (GraphPad Software Inc., La Jolla, CA, USA) was used. Statistical differences in bacterial growth and enzymatic activities were determined using the Student's *t*-test with *P* < 0.05.

## Results

AmiD^wol^ is a predicted 497 amino acid protein with 27% amino acid sequence identity with the *E. coli* AmiD sequence and contains a predicted N-terminal signal sequence at amino acids 1–30 for secretion into the periplasm (Figure 1). However, the AmiD^wol^ signal sequence lacks a lipobox motif with a conserved cysteine residue for lipidation and subsequent insertion into the outer membrane (*E. coli* AmiD “L^14^AGC^17^”; Figure 1) suggesting that AmiD^wol^ is not a lipoprotein.

### AmiD^wol^ is secreted into the *E. coli* periplasm

To test if AmiD^wol^ is transported into the periplasm after expression, the protein was overproduced with its native N-terminal signal sequence in *E. coli* JM83 using the expression vector pASK-IBA3plus. The cells were harvested and the outer membrane and cell wall were disrupted by the antibiotic polymyxin B and lysozyme, respectively, to release the periplasmic proteins. Western Blot analysis confirmed the presence of the soluble AmiD^wol^ in the periplasmic fraction (Supplementary Figure 1).

### AmiD^wol^ is functional *In vitro* and *In vivo*

For functional analysis of AmiD^wol^, the enzyme was produced in *E. coli* by co-solvent assisted periplasmic expression as previously established for chlamydial proteins (Otten et al., 2015). For this, the native signal sequence of *amiD* was replaced with the *ompA* signal sequence of the expression vector pASK-IBA2C and mannitol was used as co-solvent for protein stabilization.

**Figure 2:**
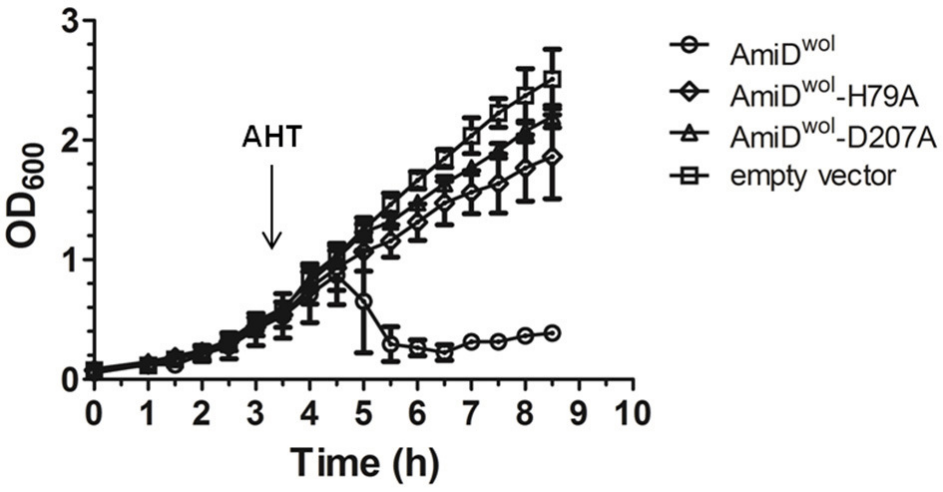
AmiD^wol^ expressed in *E. coli* has *in vivo* activity. Growth kinetics of *E. coli* JM83 containing expression vectors for periplasmic targeting of proteins. Protein expression was induced at an OD_600_ of 0.6 with anhydrotetracycline (AHT). Periplasmic overexpression of AmiD^wol^ resulted in lysis of host cells compared to the amidase active-site mutants AmiD^wol^-H79A and AmiD^wol^-D207A and the empty vector control. OD_600_ was measured every 30 min for 8.5 h. Each point represents mean ± SD. The graph is representative of three experiments.

When AmiD^wol^ was overexpressed by addition of AHT, the turbidity of the *E. coli* culture decreased gradually, indicating AmiD^wol^ hydrolytic activity on PG and subsequent lysis of the host cells. In contrast, cells carrying the empty vector pASK-IBA2C or the plasmid for the expression of an inactive version of AmiD (AmiD^wol^-H79A or AmiD^wol^-D207A in which one of the zinc-coordinating residues was replaced by alanine) did not show any growth defects (Figure 2).

**Figure 3:**
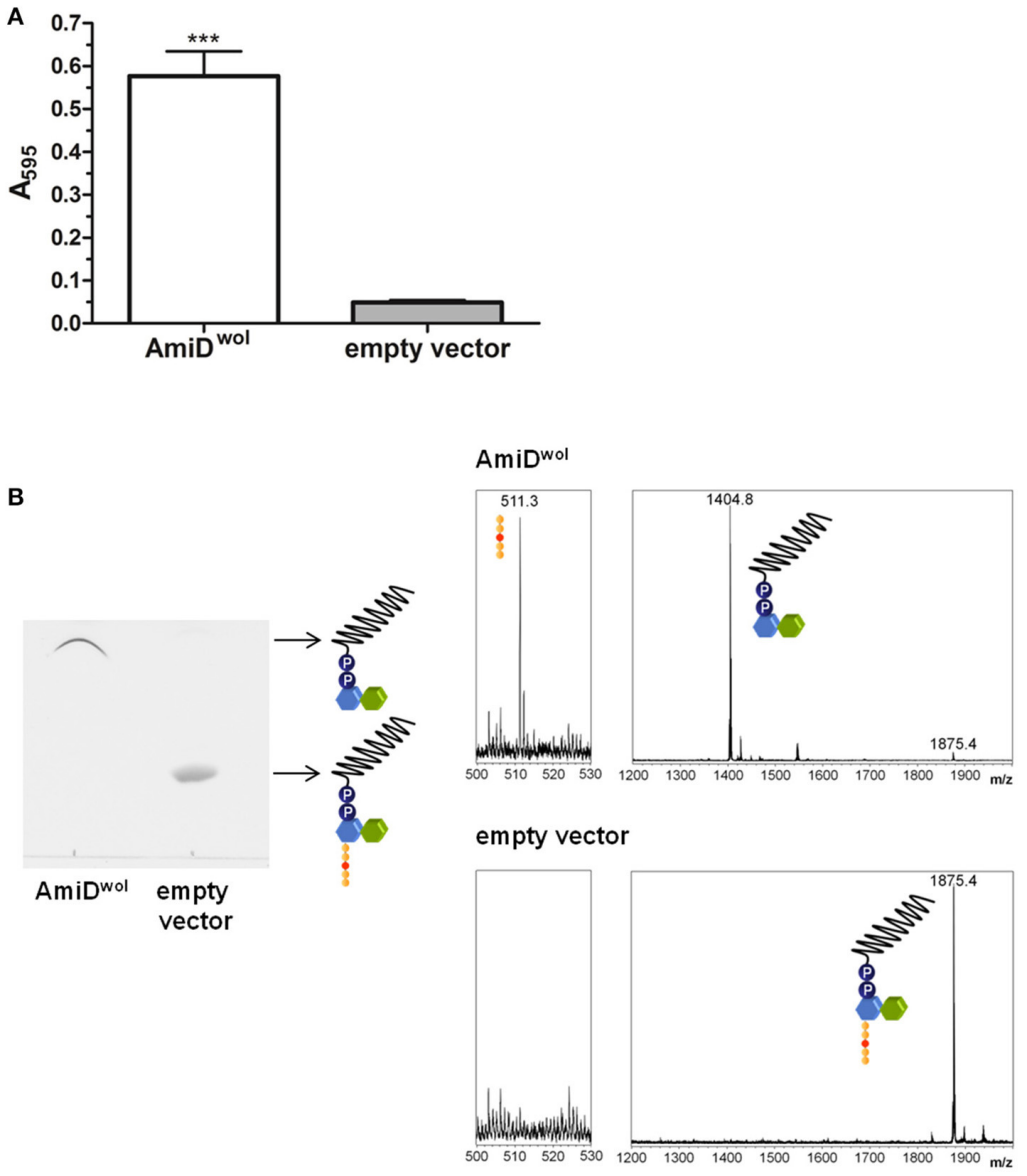
AmiD^wol^ can use PG and lipid II as substrates *in vitro*. **(A)** Degradation of PG was detected by monitoring the absorbance at 595 nm of Remazol Brilliant Blue dye released into the supernatant after incubation with AmiD^wol^ overnight at 30°C. Product from cells containing an empty expression vector was used as a negative control. Bars indicate mean ± SEM. The graph is representative of six experiments with different batches of purified enzyme. **(B)**. Lipid II was incubated with AmiD^wol^ for 4 h at 30°C. The reaction products were analyzed by TLC and MS (m/z—lipid II: 1,875.4; undecaprenyl-pyrophosphoryl-Mur*N*Ac-Glc*N*Ac: 1,404.8; pentapeptide (sodium adduct): 511.3). Statistical difference was determined using student's *t*-test, with *P* < 0.05. ^***^P < 0.001.

AmiD^wol^ was purified by Strep-tag affinity chromatography. The activity of purified AmiD^wol^ was analyzed in a dye-release assay by incubating the enzyme with RBB-stained PG from *E. coli*. Released reaction products in the supernatant resulting from PG cleavage were quantified by absorbance measurement (Figure 3A). Recombinant AmiD^wol^ was fully active at pH 7-9 and temperatures ranging from 20 to 37°C (Supplementary Figure 2). Hence, all further activity tests were performed at 30°C and at pH 7.5.

We next tested whether AmiD^wol^ can remove the pentapeptide from the cell wall precursor lipid II. Purified lipid II was incubated with AmiD^wol^ and the reaction products were extracted and analyzed by TLC and MS. In contrast to *E. coli* AmiD (Pennartz et al., 2009), AmiD^wol^ was able to use lipid II as a substrate and hydrolyzed the amide bond between Mur*N*Ac and L-Ala (Figure 3B).

**Figure 4:**
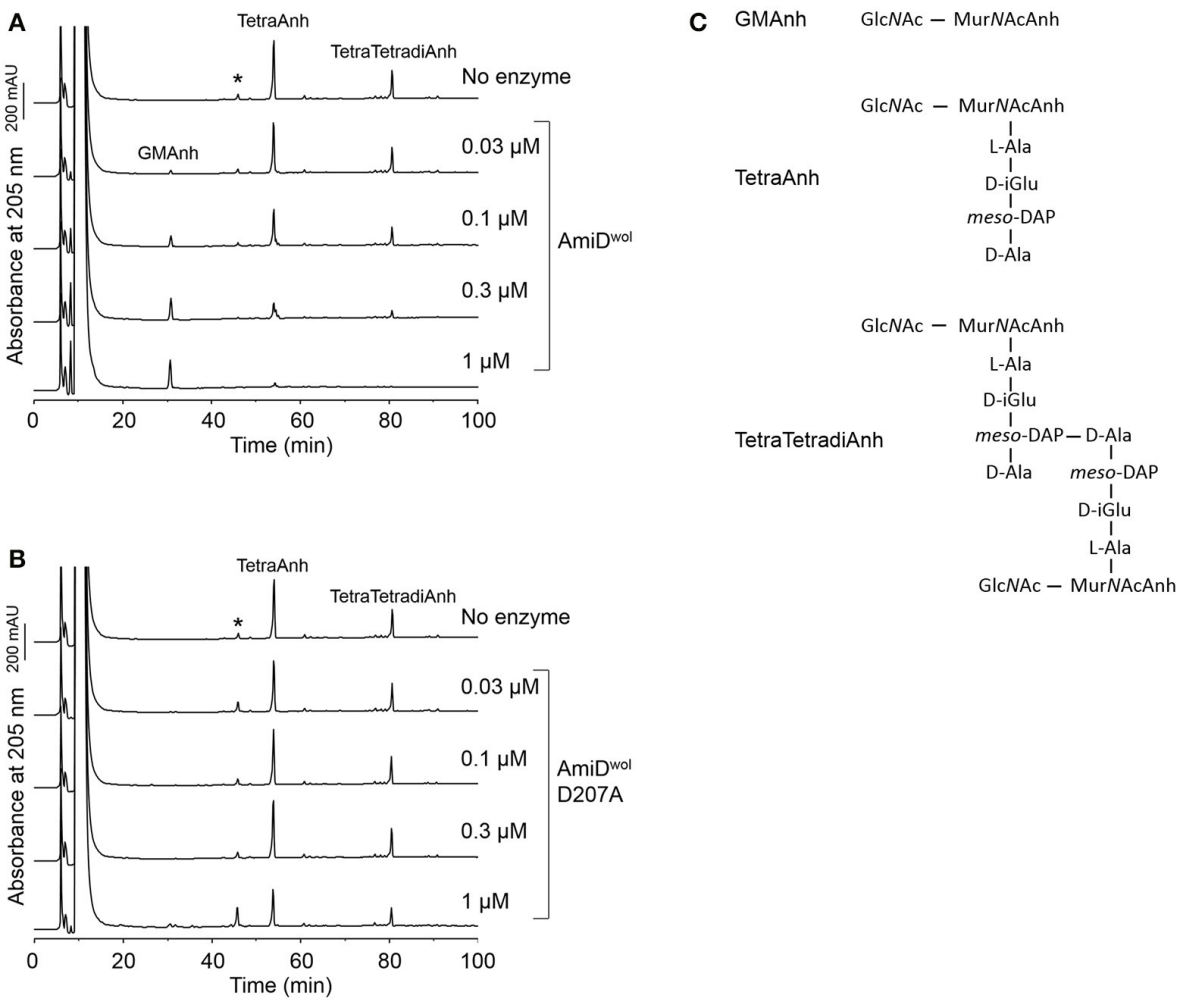
AmiD^wol^ can use anhydromuropeptides as substrates. **(A)** Anhydromuropeptides (TetraAnh, TetraTetradiAnh) derived from PG digested with the *E. coli* lytic transglyosylase Slt70 were incubated for 4 h at 30°C with AmiD^wol^ or **(B)** the amidase active-site mutant AmiD^wol^- D207A at different concentrations. The samples were analyzed by HPLC and MS. The main product was identified as Glc*N*Ac-Mur*N*AcAnh (m/z = 479.1866, H^+^ form; theoretical value: 479.1877). **(C)** Structures of the muropeptides and the reaction products analyzed in **(A,B)**. ^*^Unknown compound unrelated to the reaction.

*E. coli* AmiD has a broad substrate specificity and can also cleave anhydroMur*N*Ac-L-Ala-bonds produced by lytic transglycosylases during cell growth (Uehara and Park, 2007). Therefore, TetraAnh and TetraTetradiAnh (Figure 4C) resulting from PG digested with the *E. coli* lytic transglycosylase Slt70 were incubated with AmiD^wol^. The products were separated by HPLC and confirmed by MS. AmiD^wol^ hydrolyzed TetraAnh and TetraTetradiAnh in a dose dependent manner (Figure 4A), whereas the mutant protein AmiD^wol^-D207A (in which one of the zinc-coordinating residues was replaced by alanine) was inactive (Figure 4B).

Together, these data demonstrate that AmiD^wol^ is capable of cleaving intact PG, the cell wall precursor lipid II, as well as soluble PG fragments including the anhydro form.

### AmiD^wol^ activity is zinc-dependent

**Figure 5:**
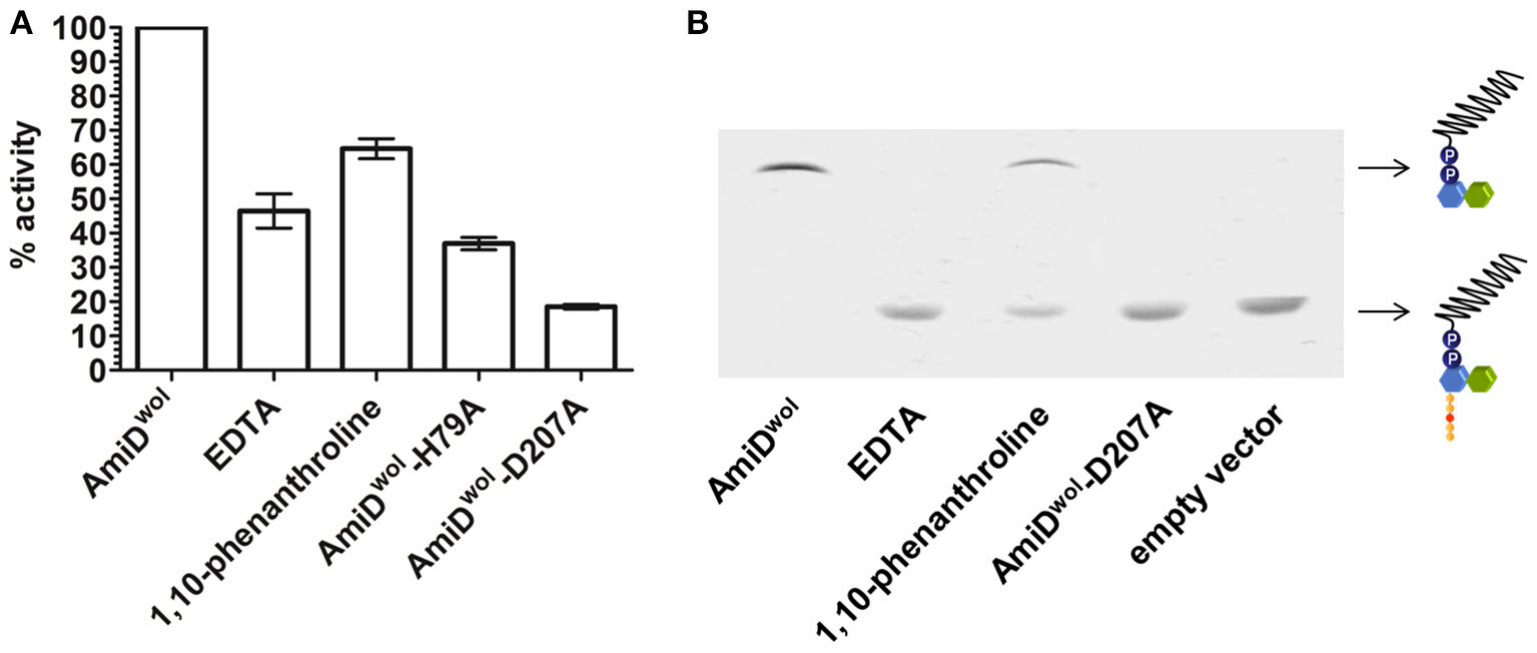
Inhibition of AmiD^wol^ activity. The zinc-dependent amidase activity could be inhibited in presence of 1 mM of the non-specific metal chelator EDTA and 5 mM of the Zn^2+^-specific chelator 1,10-phenanthroline as shown by **(A)** Remazol Brilliant Blue dye-release assay and **(B)** thin layer chromatography. Moreover, the exchange of one of the zinc-coordinating residues with alanine (H79A or D207A) decreased amidase activity. Results shown are means ± SEM. The graph is representative of three experiments with different batches of purified enzyme.

The active site of AmiD^wol^ is comprised of three conserved zinc-coordinating residues (H79, H187, D207; Figure 1 and Supplementary Figure 3) that are essential for amidase activity (Uehara and Park, 2007; Kerff et al., 2010). PG and lipid II cleavage by AmiD^wol^ was decreased in the presence of EDTA and the specific Zn^2+^-chelator 1,10-phenanthroline (Figure 5A), although the enzyme activity was not completely inhibited by either compound, as has been shown for the *E. coli* ortholog (Uehara and Park, 2007). The mutation of one residue involved in zinc binding (H79A or D207A) resulted in a loss of activity, with AmiD^wol^-H79A and AmiD^wol^-D207A unable to hydrolyze the Mur*N*Ac-L-Ala bond in PG and lipid II (Figures 4B, 5B). Expression of the amidase active-site mutants AmiD^wol^-H79A and AmiD^wol^-D207A in *E. coli* also did not induce cell lysis *in vivo* (Figure 2).

## Discussion

*Wolbachia* are obligate intracellular bacteria with highly reduced genomes due to the adaption to- and dependency on their hosts (Moran and Plague, 2004). Interestingly, the genome of arthropod *Wolbachia* such as *w*Mel encodes a peptidoglycan hydrolase (Wu et al., 2004), homologous to the Mur*N*Ac-L-Ala amidase AmiD, although a functional cell wall has not been detected.

In this study we characterized AmiD^wol^ to understand its role in *Wolbachia* biology. The presence of an N-terminal signal peptide predicts periplasmic localization of AmiD^wol^. Our results demonstrate that the wolbachial periplasmic signal sequence can substitute for the OmpA leader peptide and that AmiD^wol^ is indeed exported into the periplasm (Supplementary Figure 1). In contrast to the *E. coli* homolog, the AmiD^wol^ signal peptide does not include a cysteine-containing lipobox motif for further modification and insertion into the outer membrane (Figure 1). Thus, AmiD^wol^ is probably transported to the periplasm by the Sec pathway and cleaved between residues 30 and 31. The only *Wolbachia* lipoproteins identified from *w*Bm and insect *Wolbachia pipientis* strain *A. albopictus* B (*w*AlbB) to date are the peptidoglycan associated lipoprotein (wPAL) and the type IV secretion system component VirB6 (Voronin et al., 2014). Both are probably diacylated due to the absence of the gene encoding the N-acetyltransferase Ltn.

This is the first description of a wolbachial lipid II processing enzyme putatively expressed in the periplasm. Our results demonstrate that AmiD^wol^ hydrolyzes the amide bond between Mur*N*Ac and L-Ala of various substrates as has been shown for *E. coli* AmiD. The functional conservation of AmiD^wol^ indicates that the ability to cleave anhydromuropeptides may play an important role in the lifestyle of insect *Wolbachia* and that these endobacteria may contain a PG-like structure with connected glycan strands because 1,6-anhydro bonds are generated by periplasmic lytic transglycosylases that cleave the glycosidic bond between Mur*N*Ac and Glc*N*Ac units. However, neither a typical PG glycosyltransferase that could link the sugar moieties of the cell wall building block lipid II nor a lytic transglycosylase that could catalyze glycan chain cleavage during bacterial growth have been identified in the *Wolbachia* genomes (Wu et al., 2004; Foster et al., 2005). However, it has been recently suggested that RodA, a member of the SEDS (shape, elongation, division, sporulation) protein family, can polymerize glycan strands in the absence of known PG glycosyltransferases in *Bacillus subtilis* (Meeske et al., 2016). The absence of these enzymes in intracellular *Chlamydia, Planctomycetes*, and *Wolbachia* suggests that monofunctional PG transpeptidase-SEDS pairs might have been retained as principal PG polymerase systems (Henrichfreise et al., 2016). Supporting this hypothesis, recent studies demonstrate that PG can be detected and extracted from *Chlamydia* and *Planctomycetes* (Pilhofer et al., 2013; Liechti et al., 2014; Jeske et al., 2015; van Teeseling et al., 2015). It remains to be seen if the RodA/FtsW ortholog in *Wolbachia* has such an activity for the assembly of PG together with a monofunctional transpeptidase.

AmiA from *Chlamydia pneumoniae* has been demonstrated to rescue cell division in an *E. coli* strain deficient for AmiA, AmiB, and AmiC (Klöckner et al., 2014). *E. coli* AmiD was shown not to be involved in cell separation (Uehara and Park, 2007), therefore we would expect a similar result when using AmiD^wol^ expressed in *E. coli* deficient for the other amidases. Attempts were made to rescue such mutants, however expressed AmiD^wol^ was half the expected size and no complementation was seen (data not shown). We cannot rule out that AmiD^wol^ is involved in the turnover of a rudimentary PG-like structure as the recycling pathway of C_55_-P remains unclear (Henrichfreise et al., 2009; Vollmer et al., 2013). The exact role of AmiD^wol^ in lipid II/PG modification is still undefined. This raises the question why only AmiD is conserved in *Wolbachia* that behave more as parasites in arthropods.

In contrast to all other amidases in *E. coli*, AmiD has a broad substrate specificity and its role is still unclear. Uehara and Park (2007) proposed that the breakdown of cell wall fragments in the periplasm by AmiD is a secondary strategy to prevent immune responses in the host. *Drosophila spp*. relies entirely on innate immunity and two pathways respond to different classes of microorganisms (Buchon et al., 2014). The Toll pathway is mainly activated in response to Gram-positive bacteria and fungi, whereas the immune deficiency (Imd) pathway is mostly triggered by Gram-negative bacteria. Recognition of bacteria is mostly achieved by PG recognition proteins (PGRPs). The membrane anchored receptor PGRP-LC and the cytoplasmic receptor PGRP-LE upstream of the Imd pathway sense PG fragments containing mDAP in the peptide side chains found in Gram-negative bacteria and the Gram-positive bacilli (Buchon et al., 2014; Myllymaki et al., 2014). The minimum structure for recognition by PGRP-LC is a monomer of Glc*N*Ac-Mur*N*Ac with an internal 1,6-anhydro-bond attached to a tripeptide (Stenbak et al., 2004). Thus, in addition to a possible role in processing lipid II for recycling, AmiD^wol^ might also help to suppress host immune responses by removing the peptide chain from the sugar moieties. Because insect *Wolbachia* are parasites and can horizontally infect other insects (Werren et al., 2008), this enzyme may have been maintained for example in *w*Mel, *Wolbachia* from *Drosophila simulans* (*w*Ri) and *Wolbachia* from *Culex quinquefasciatus* Pel (*w*Pip) (Figure 1) to aid this specific endosymbiotic lifestyle and protect these endobacteria. In contrast, genomes of sequenced mutualistic *Wolbachia* from filarial nematodes (Foster et al., 2005) and the bedbug *C. lectularis* (Nikoh et al., 2014) show that these strains have lost the ability to synthesize AmiD^wol^. Moreover, nematodes do not express orthologs of PGRP-LC or Imd and thus would not recognize the same PG metabolism/recycling products, allowing *Wolbachia* of filarial nematodes to lose AmiD^wol^ during evolution as mutualistic endosymbionts (Irazoqui et al., 2010; Ermolaeva and Schumacher, 2014). This hypothesis of AmiD as a protective mechanism will benefit from comparative analysis of *Wolbachia* genomes from all supergroups.

## Author contributions

AH and KP conceptualized the study and obtained funding. MW, KM, AS, AK, BH, and KP conceived and designed the experiments. MW, KM, MJ, AK, and CO performed the laboratory experiments and analyzed data. MW, KM, and KP drafted the manuscript, with critical review by AH and WV.

### Conflict of interest statement

The authors declare that the research was conducted in the absence of any commercial or financial relationships that could be construed as a potential conflict of interest.

## References

[B1] Buchon N. Silverman N. Cherry S. (2014). Immunity in *Drosophila melanogaster* from microbial recognition to whole-organism physiology. Nat. Rev. Immunol. 14, 796–810. 10.1038/nri3763 25421701 PMC6190593

[B2] Bui N. K. Gray J. Schwarz H. Schumann P. Blanot D. Vollmer W. (2009). The peptidoglycan sacculus of *Myxococcus xanthus* has unusual structural features and is degraded during glycerol-induced myxospore development. J. Bacteriol. 191, 494–505. 10.1128/JB.00608-08 18996994 PMC2620817

[B3] Ermolaeva M. A. Schumacher B. (2014). Insight from the worm: the *C. elegans* model for innate immunity. Semin. Immunol. 26, 303–309. 10.1016/j.smim.2014.04.005 24856329 PMC4248339

[B4] Foster J. Ganatra M. Kamal I. Ware J. Makarova K. Ivanova N.. (2005). The *Wolbachia* genome of *Brugia malayi*: endosymbiont evolution within a human pathogenic nematode. PLoS Biol. 3:e121. 10.1371/journal.pbio.0030121 15780005 PMC1069646

[B5] Glauner B. (1988). Separation and quantification of muropeptides with high-performance liquid-chromatography. Anal. Biochem. 172, 451–464. 10.1016/0003-2697(88)90468-X 3056100

[B6] Heidrich C. Templin M. F. Ursinus A. Merdanovic M. Berger J. Schwarz H.. (2001). Involvement of N-acetylmuramyl-L-alanine amidases in cell separation and antibiotic-induced autolysis of *Escherichia coli*. Mol. Microbiol. 41, 167–178. 10.1046/j.1365-2958.2001.02499.x 11454209

[B7] Henrichfreise B. Brunke M. Viollier P. H. (2016). Bacterial surfaces: the wall that SEDS built. Curr. Biol. 26, R1158–R1160. 10.1016/j.cub.2016.09.028 27825456

[B8] Henrichfreise B. Schiefer A. Schneider T. Nzukou E. Poellinger C. Hoffmann T. J.. (2009). Functional conservation of the lipid II biosynthesis pathway in the cell wall-less *Chlamydia* and *Wolbachia*: why is lipid II needed? Mol. Microbiol. 73, 913–923. 10.1111/j.1365-2958.2009.06815.x 19656295

[B9] Irazoqui J. E. Urbach J. M. Ausubel F. M. (2010). Evolution of host innate defense: insights from *Caenorhabditis elegans* and primitive invertebrates. Nat. Rev. Immunol. 10, 47–58. 10.1038/nri2689 20029447 PMC2965059

[B10] Jacobs C. Joris B. Jamin M. Klarsov K. Van Beeumen J. Mengin-Lecreulx D.. (1995). AmpD, essential for both beta-lactamase regulation and cell wall recycling, is a novel cytosolic N-acetylmuramyl-L-alanine amidase. Mol. Microbiol. 15, 553–559. 10.1111/j.1365-2958.1995.tb02268.x 7783625

[B11] Jeske O. Schüler M. Schumann P. Schneider A. Boedeker C. Jogler M.. (2015). Planctomycetes do possess a peptidoglycan cell wall. Nat. Commun. 6:7116. 10.1038/ncomms8116 25964217 PMC4432640

[B12] Johnson J. W. Fisher J. F. Mobashery S. (2013). Bacterial cell-wall recycling. Ann. N.Y. Acad. Sci. 1277, 54–75. 10.1111/j.1749-6632.2012.06813.x 23163477 PMC3556187

[B13] Kerff F. Petrella S. Mercier F. Sauvage E. Herman R. Pennartz A.. (2010). Specific structural features of the N-acetylmuramoyl-L-alanine amidase AmiD from *Escherichia coli* and mechanistic implications for enzymes of this family. J. Mol. Biol. 397, 249–259. 10.1016/j.jmb.2009.12.038 20036252

[B14] Klöckner A. Otten C. Derouaux A. Vollmer W. Bühl H. De Benedetti S.. (2014). AmiA is a penicillin target enzyme with dual activity in the intracellular pathogen *Chlamydia pneumoniae*. Nat. Commun. 5:4201. 10.1038/ncomms5201 24953137 PMC4083426

[B15] Lenz J. D. Stohl E. A. Robertson R. M. Hackett K. T. Fisher K. Xiong K.. (2016). Amidase activity of AmiC controls cell separation and stem peptide release and is enhanced by NlpD in *Neisseria gonorrhoeae*. J. Biol. Chem. 291, 10916–10933. 10.1074/jbc.M116.715573 26984407 PMC4865936

[B16] Liechti G. W. Kuru E. Hall E. Kalinda A. Brun Y. V. VanNieuwenhze M.. (2014). A new metabolic cell-wall labelling method reveals peptidoglycan in *Chlamydia trachomatis*. Nature 506, 507–510. 10.1038/nature12892 24336210 PMC3997218

[B17] Lindsey A. R. Werren J. H. Richards S. Stouthamer R. (2016). Comparative genomics of a parthenogenesis-inducing *Wolbachia* symbiont. G3 6, 2113–2123. 10.1534/g3.116.028449 27194801 PMC4938664

[B18] Meeske A. J. Riley E. P. Robins W. P. Uehara T. Mekalanos J. J. Kahne D.. (2016). SEDS proteins are a widespread family of bacterial cell wall polymerases. Nature 537, 634–638. 10.1038/nature19331 27525505 PMC5161649

[B19] Möll A. Dörr T. Alvarez L. Chao M. C. Davis B. M. Cava F. (2014). Cell separation in *Vibrio cholera* is mediated by a single amidase whose action is modulated by two nonredundant activators. J. Bacteriol. 196, 3937–3948. 10.1128/JB.02094-14 25182499 PMC4248829

[B20] Moran N. A. Plague G. R. (2004). Genomic changes following host restriction in bacteria. Curr. Opin. Genet. Dev. 14, 627–633. 10.1016/j.gde.2004.09.003 15531157

[B21] Myllymaki H. Valanne S. Ramet M. (2014). The *Drosophila* imd signaling pathway. J. Immunol. 192, 3455–3462. 10.4049/jimmunol.1303309 24706930

[B22] Nikoh N. Hosokawa T. Moriyama M. Oshima K. Hattori M. Fukatso T. (2014). Evolutionary origin of insect-*Wolbachia* nutritional mutualism. Proc. Natl. Acad. Sci. U.S.A. 111, 10257–10262. 10.1073/pnas.1409284111 24982177 PMC4104916

[B23] Otten C. De Benedetti S. Gaballah A. Bühl H. Klöckner A. Brauner J.. (2015). Co-solvents as stabilizing agents during heterologous overexpression in *Escherichia coli*- application to chlamydial penicillin-binding protein 6. PLoS ONE 10:e0122110. 10.1371/journal.pone.0122110 25849314 PMC4388811

[B24] Pennartz A. Genereux C. Parquet C. Mengin-Lecreulx D. Joris B. (2009). Substrate-induced inactivation of the *Escherichia coli* AmiD N-Acetylmuramoyl-L-Alanine amidase highlights a new strategy to inhibit this class of enzyme. Antimicrob. Agents Chemother. 53, 2991–2997. 10.1128/AAC.01520-07 19237650 PMC2704666

[B25] Pilhofer M. Aistleitner K. Biboy J. Gray J. Kuru E. Hall E.. (2013). Discovery of chlamydial peptidoglycan reveals bacteria with murein sacculi but without FtsZ. Nat. Commun. 4:2856. 10.1038/ncomms3856 24292151 PMC3847603

[B26] Priyadarshini R. de Pedro M. A. Young K. D. (2007). Role of peptidoglycan amidases in the development and morphology of the division septum in *Escherichia coli*. J. Bacteriol. 189, 5334–5347. 10.1128/JB.00415-07 17483214 PMC1951850

[B27] Rouser G. Fleischer S. Yamamoto A. (1970). Two dimensional then layer chromatographic separation of polar lipids and determination of phospholipids by phosphorus analysis of spots. Lipids 5, 494–496. 10.1007/BF02531316 5483450

[B28] Schneider T. Senn M. M. Berger-Bachi B. Tossi A. Sahl H. G. Wiedemann I. (2004). *In vitro* assembly of a complete, pentaglycine interpeptide bridge containing cell wall precursor (lipid II-Gly5) of *Staphylococcus aureus*. Mol. Microbiol. 53, 675–685. 10.1111/j.1365-2958.2004.04149.x 15228543

[B29] Specht S. Debrah A. Y. Klarmann U. Mand S. Hoerauf A. Pfarr K. (2013). Chemotherapy of filariasis - established strategies and new developments. GMS Infect. Dis. 1, 1–10. 10.3205/id000003

[B30] Stenbak C. R. Ryu J. H. Leulier F. Pili-Floury S. Parquet C. Herve M.. (2004). Peptidoglycan molecular requirements allowing detection by the *Drosophila* immune deficiency pathway. J. Immunol. 173, 7339–7348. 10.4049/jimmunol.173.12.7339 15585858

[B31] Stepkowski T. Legocki A. B. (2001). Reduction of bacterial genome size and expansion resulting from obligate intracellular lifestyle and adaptation to soil habitat. Acta Biochim. Pol. 48, 367–381. 11732608

[B32] Taylor M. J. Hoerauf A. (2001). A new approach to the treatment of filariasis. Curr. Opin. Infect. Dis. 14, 727–731. 10.1097/00001432-200112000-00011 11964892

[B33] Taylor M. J. Hoerauf A. Bockarie M. (2010). Lymphatic filariasis and onchocerciasis. Lancet 376, 1175–1185. 10.1016/S0140-6736(10)60586-7 20739055

[B34] Typas A. Banzhaf M. Gross C. A. Vollmer W. (2012). From the regulation of peptidoglycan synthesis to bacterial growth and morphology. Nat. Rev. Microbiol. 10, 123–136. 10.1038/nrmicro2677 22203377 PMC5433867

[B35] Uehara T. Dinh T. Bernhardt T. G. (2009). LytM-domain factors are required for daughter cell separation and rapid ampicillin-induced lysis in *Escherichia coli*. J. Bacteriol. 191, 5094–5107. 10.1128/JB.00505-09 19525345 PMC2725582

[B36] Uehara T. Park J. T. (2007). An anhydro-N-acetylmuramyl-L-alanine amidase with broad specificity tethered to the outer membrane of *Escherichia coli*. J. Bacteriol. 189, 5634–5641. 10.1128/JB.00446-07 17526703 PMC1951811

[B37] Uehara T. Parzych K. R. Dinh T. Bernhardt T. G. (2010). Daughter cell separation is controlled by cytokinetic ring-activated cell wall hydrolysis. EMBO J. 29, 1412–1422. 10.1038/emboj.2010.36 20300061 PMC2868575

[B38] van Teeseling M. C. Mesman R. J. Kuru E. Espaillat A. Cava F. Brun Y. V.. (2015). Annamox planctomycetes have a peptidoglycan cell wall. Nat. Commun. 6:6878. 10.1038/ncomms7878 25962786 PMC4432595

[B39] Vollmer J. Schiefer A. Schneider T. Jülicher K. Johnston K. L. Taylor M. J.. (2013). Requirement of lipid II biosynthesis for cell division in cell wall-less *Wolbachia*, endobacteria of arthropods and filarial nematodes. Int. J. Med. Microbiol. 303, 140–149. 10.1016/j.ijmm.2013.01.002 23517690

[B40] Vollmer W. Joris B. Charlier P. Foster S. (2008). Bacterial peptidoglycan (murein) hydrolases. FEMS Microbiol. Rev. 32, 259–286. 10.1111/j.1574-6976.2007.00099.x 18266855

[B41] Voronin D. Guimaraes A. F. Molyneux G. R. Johnston K. L. Ford L. Taylor M. J. (2014). Wolbachia lipoproteins: abundance, localisation and serology of *Wolbachia* peptidoglycan associated lipoprotein and the type IV secretion system component, VirB6 from *Brugia malayi* and *Aedes albopictus*. Parasit. Vectors 7:462. 10.1186/s13071-014-0462-1 25287420 PMC4197220

[B42] Werren J. H. Baldo L. Clark M. E. (2008). *Wolbachia*: master manipulators of invertebrate biology. Nat. Rev. Microbiol. 6, 741–751. 10.1038/nrmicro1969 18794912

[B43] Wu M. Sun L. V. Vamathevan J. Riegler M. Deboy R. Brownlie J. C.. (2004). Phylogenomics of the reproductive parasite *Wolbachia pipientis w*Mel: a streamlined genome overrun by mobile genetic elements. PLoS Biol. 2:E69. 10.1371/journal.pbio.0020069 15024419 PMC368164

[B44] Zug R. Hammerstein P. (2015). Bad guys turned nice? A critical assessment of Wolbachia mutualism in arthropod hosts. Biol. Rev. Camb. Philos. Soc. 90, 89–111. 10.1111/brv.12098 24618033

