# Supplementary material for: AmiD Is a Novel Peptidoglycan Amidase in Wolbachia Endosymbionts of Drosophila melanogaster
Source: Front Cell Infect Microbiol. 2017 Aug 4;7:353. doi: 10.3389/fcimb.2017.00353 (PMC5543032; doi:10.3389/fcimb.2017.00353)
Supplement: Supplementary file 1 [file DataSheet1.DOCX]

Supplementary Material

AmiD is a novel peptidoglycan amidase in *Wolbachia* endosymbionts of *Drosophila melanogaster*

Miriam Wilmes^#1^, Kirstin Meier^#1^, Andrea Schiefer^1^, Michaele Josten^1^, Christian Otten^2^, Anna Klöckner^3,4^, Beate Henrichfreise^3^, Waldemar Vollmer^2^, Achim Hoerauf^1, 4^*, Kenneth Pfarr^1^*

^#^ contributed equally to this work

*** Correspondence:** Achim.Hoerauf@ukbonn.de, Kenneth.Pfarr@ukbonn.de

# Supplementary Figures


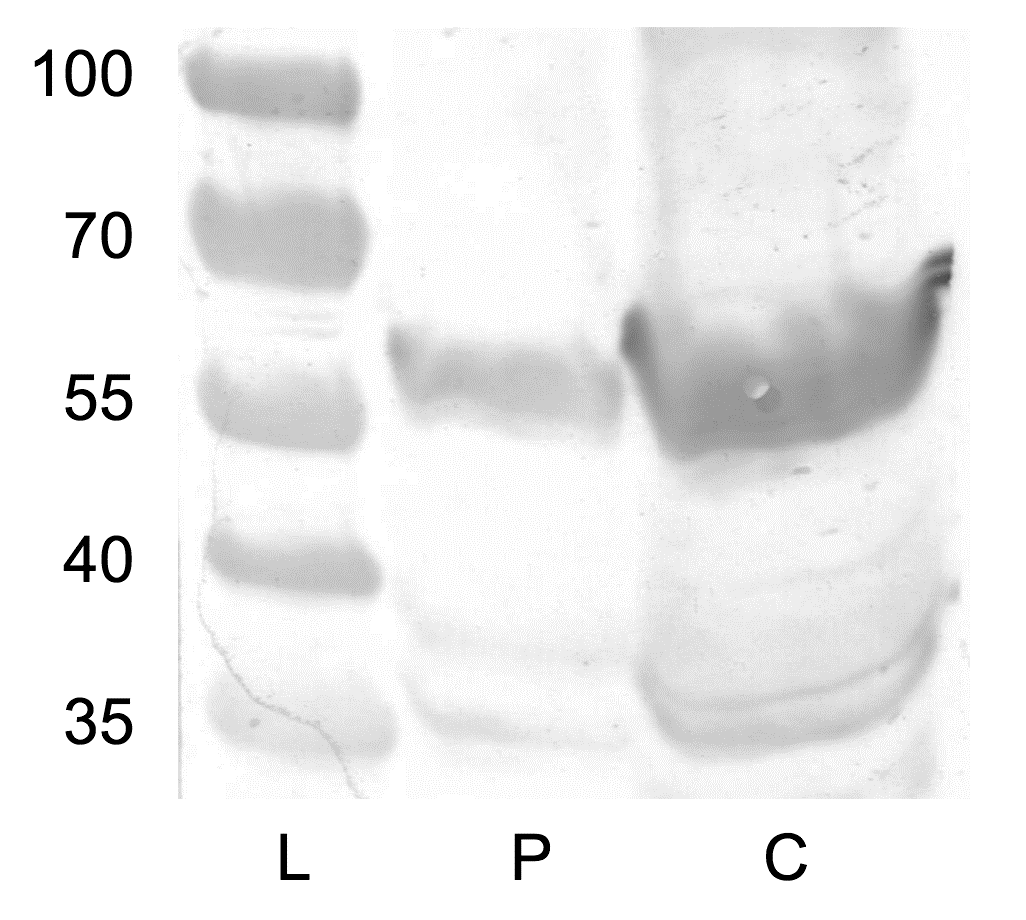


**Figure 1: Cellular distribution of AmiD^wol^ after cytoplasmic expression in *E. coli*.** Western Blot analysis confirmed the presence of AmiD^wol^ in the periplasm after expression. The outer membrane and cell wall were disrupted with polymyxin B and lysozyme. The treated cells were centrifuged at 18,000 g at 4 °C to separate the periplasmic contents (found in the supernatant) from cytoplasmic contents (found in the pellet). The different fractions were separated by 12% SDS-PAGE and detected using Strep-Tactin Alkaline Phosphatase conjugate. The picture is representative of three expressions experiments. L: Ladder, P: Periplasmic fraction, C: Cytoplasmic Fraction.


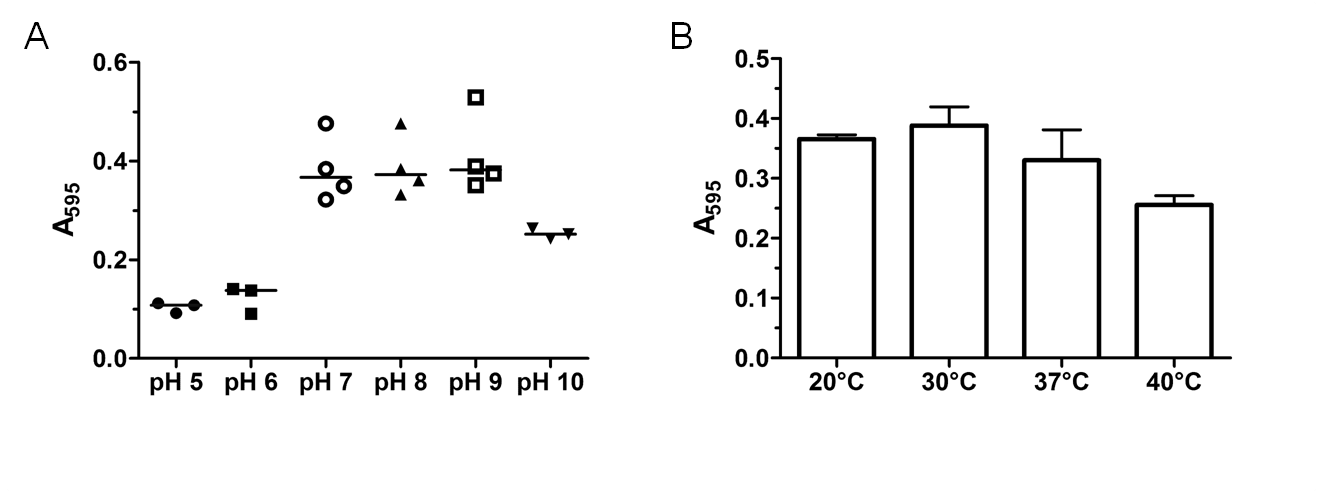


**Figure 2: Activity of AmiD^wol^ at different pH and temperatures.** To find the optimal conditions for amidase activity, degradation of PG was measured by monitoring the absorbance at 595 nm of Remazol Brilliant Blue dye released into the supernatant after incubation with AmiD^wol^ overnight at 30 °C. Results show the mean ± SEM (n=3-4).

**
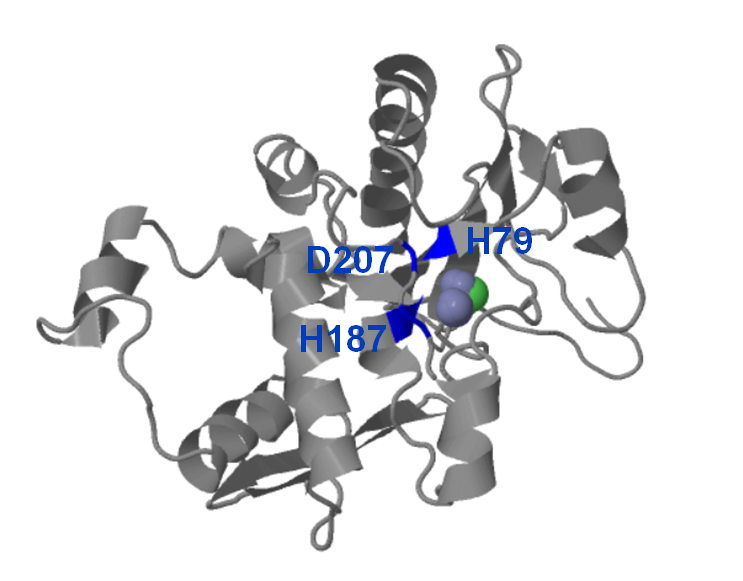
**

**Figure 3: 3D structure of AmiD^wol^ predicted by Phyre^2^** (<http://www.sbg.bio.ic.ac.uk/phyre2/html/page.cgi?id=index>). The zinc-coordinating residues H79, H187 and D207 are marked in blue, zinc ions are marked in purple.

**
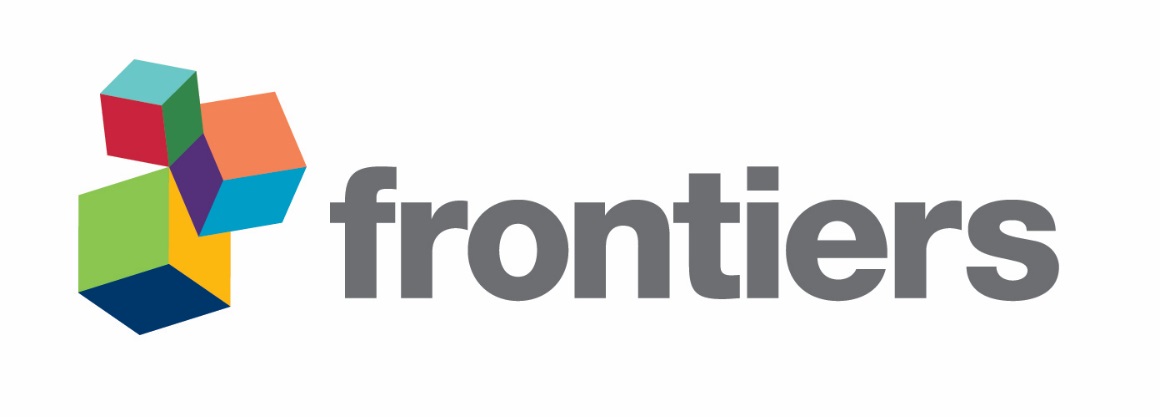
**
